# In-Frame 12-Nucleotide Deletion within Open Reading Frame 3a in a SARS-CoV-2 Strain Isolated from a Patient Hospitalized with COVID-19

**DOI:** 10.1128/MRA.00137-21

**Published:** 2021-02-25

**Authors:** John A. Lednicky, Kartikeya Cherabuddi, Massimiliano S. Tagliamonte, Maha A. Elbadry, Kuttichantran Subramaniam, Thomas B. Waltzek, J. Glenn Morris

**Affiliations:** a Emerging Pathogens Institute, University of Florida, Gainesville, Florida, USA; b Department of Environmental and Global Health, College of Public Health and Health Professions, University of Florida, Gainesville, Florida, USA; c Department of Medicine, College of Medicine, University of Florida, Gainesville, Florida, USA; d Department of Pathology, Immunology, and Laboratory Medicine, College of Medicine, University of Florida, Gainesville, Florida, USA; e Department of Infectious Diseases and Immunology, College of Veterinary Medicine, University of Florida, Gainesville, Florida, USA; Queens College

## Abstract

Severe acute respiratory syndrome coronavirus 2 (SARS-CoV-2) strain UF-8, with an in-frame 12-nucleotide deletion within open reading frame 3a (ORF3a), was isolated from a 78-year-old COVID-19 patient in March 2020.

## ANNOUNCEMENT

A 78-year-old woman with COVID-19 at the University of Florida hospital’s intensive care unit in March 2020 improved clinically and was discharged 6 days later. The studies were reviewed and approved by the University of Florida Institutional Review Board (IRB).

RNA extracted from a nasopharyngeal specimen (NP) from the patient using a QIAamp viral RNA minikit (Qiagen, Valencia, CA) tested positive for severe acute respiratory syndrome coronavirus 2 (SARS-CoV-2) RNA (vRNA) using the CDC real-time reverse transcriptase PCR (rRT-PCR) diagnostic test for COVID-19 (https://www.cdc.gov/coronavirus/2019-ncov/lab/virus-requests.html), generating a quantification cycle (*C_q_*) value of 28 for primer set N1. As insufficient vRNA was extracted for direct sequencing using our standard next-generation sequencing (NGS) method, which requires a *C_q_* value of ≤20 to obtain adequate coverage, an aliquot of the NP specimen was inoculated onto Vero E6 cells in a T25 flask and incubated at 37°C for virus isolation and the preparation of larger amounts of vRNA for sequencing. The virus required 7 days to attain a virus titer of 2.5 × 10^6^ PFU/ml and induce cytopathic effects (CPEs), whereas other wild-type strains usually attain a titer of ≥3 × 10^6^ PFU/ml and induce CPEs 3 days postinoculation of the cells under the same conditions. RNA purified from the cell growth medium using a QIAamp viral RNA kit was subsequently submitted for NGS (1). Briefly, a cDNA sequencing library was made from the RNA using a NEBNext Ultra II RNA library preparation kit (New England Biolabs, Ipswich, MA). The resulting cDNA library was sequenced on a MiSeq (Illumina, San Diego, CA) instrument using a v3 600-cycle kit.

**FIG 1:**
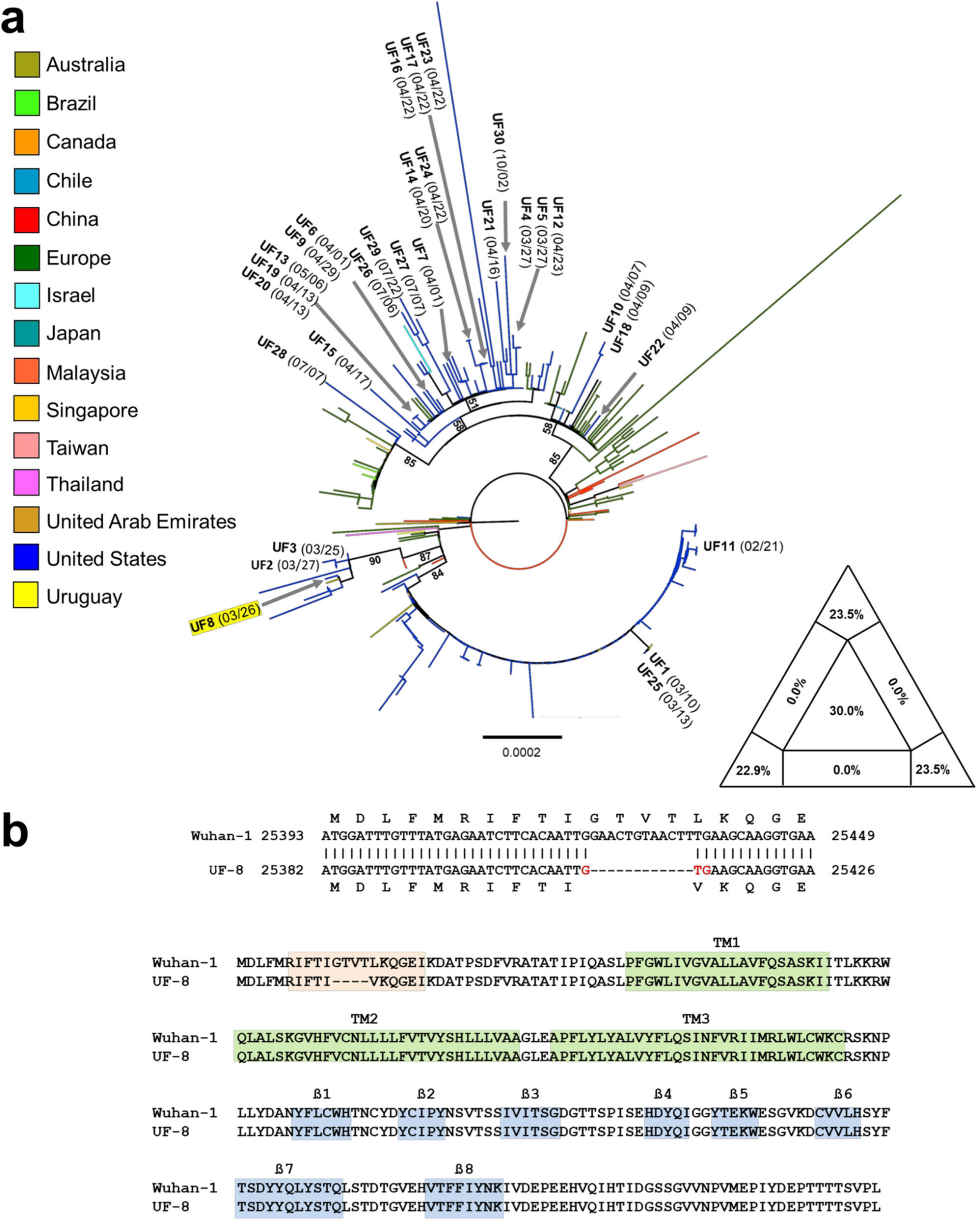
(a) SARS-CoV-2 full or nearly full genome (>29,000 bp) sequences, with a collection date prior to or on 6 March 2020, were downloaded from GISAID (https://www.gisaid.org/) on 18 August 2020. The genomes were subsequently filtered according the following exclusion criteria: (i) sequences with more than 150 uncertain nucleotides due to missing data and/or poor sequence quality, (ii) sequences missing sampling date, and (iii) sequences missing sampling location. (b) Sequence alignment of deduced 3a proteins of SARS-CoV-2 reference strain Wuhan-1 and of UF-8. Epitopes for natural antibodies against 3a are delimited by the pink box, transmembrane (TM) alpha helices (coils) are delimited by green boxes, and beta strands, by blue boxes.

A total of 2,385,756 paired-end reads were generated, and 83% (1,976,911 paired-end reads) of the Vero E6 cell host reads were removed using Kraken v2.0 with default parameters. Demultiplexing was automatically performed with the MiSeq software; no trimming was performed. *De novo* assembly of 408,845 reads (with an average read length of 190 bp) in CLC Genomics Workbench v20.0.3, with default parameters, recovered the near-complete genome sequence, 29,847 nucleotides (nt) with a G+C content of 38% excluding the first 11 nt at the 5′ end and poly(A) tail. The virus was designated strain UF-8. The genomic sequence of UF-8 was confirmed by repeating the NGS process and by site-directed RT-PCR and Sanger sequencing of RNA extracted directly from the NP swab to confirm the 12-nt deletion. Phylogenetic analyses performed as previously described (2) revealed that UF-8 was closely related to early SARS-CoV-2 strains that circulated locally, but we did not see evidence of dissemination based on sequence analyses of subsequent strains from our institution (Fig. 1a).

Importantly, the 12-nucleotide in-frame deletion occurs within open reading frame 3a (ORF3a), which encodes viroporin 3a (Fig. 1b). Viroporins are hydrophobic proteins that are considered virulence factors that are typically not essential for virus replication. However, some form pores that facilitate ion transport across cell membranes, ensuring virus release with the potential for coincident inflammasome activation (3, 4). Nevertheless, we did not see clear evidence of decreased clinical virulence caused by SARS-CoV-2 UF-8.

After filtering, 2,452 genomes, including 30 new UF isolates (UF1 to UF30), were retained and aligned using MAFFT v7.407 (5). The aligned genomes were ranked by similarity by calculating pairwise Jukes-Cantor (JC) distances. Genomes identical to UF sequences were removed from the set, and the remaining ones were randomly subsampled using the following constraints: (i) the final data set should include a minimum of 250 and maximum of 300 sequences, in addition to our UF sequences, and (ii) the median genetic diversity of the subsample should be the same as the median of the full data set. The subsampled data set, representative of the overall diversity of the full data set, included 272 sequences, plus our 30 UF sequences, for a total of 302 strains. The resulting alignment was used to infer a maximum likelihood tree with the general time-reversable (GTR) substitution model and 1,000 bootstrap replicates with IQ-TREE v2.0.6 (6), using the ModelFinder option to select the best-fitting substitution model (7). The presence of sufficient tree-like signal in the subsampled data set was assessed by likelihood mapping (8), also implemented in IQ-TREE.

### Data availability.

The complete coding DNA sequence (CDS) of SARS-CoV-2/human/USA/UF-8/2020 has been deposited in GenBank under accession number MW221275.1. The SRA accession number for the raw data is SRR13091823.
